# Characterization of a time-resolved electron microscope with a Schottky field emission gun

**DOI:** 10.1063/4.0000034

**Published:** 2020-10-01

**Authors:** Pavel K. Olshin, Marcel Drabbels, Ulrich J. Lorenz

**Affiliations:** Laboratory of Molecular Nanodynamics, École Polytechnique Fédérale de Lausanne, 1015 Lausanne, Switzerland

## Abstract

The rapid growth of the field of time-resolved and ultrafast electron microscopy has been accompanied by the active development of new instrumentation. Recently, time-resolved microscopes equipped with a field emission gun have been introduced, demonstrating great potential for experiments that benefit from the high brightness and coherence of the electron source. Here, we describe a straightforward design of a time-resolved transmission electron microscope with a Schottky field emission gun and characterize its performance. At the same time, our design gives us the flexibility to alternatively operate the instrument as if it was equipped with a flat metal photocathode. We can, thus, effectively choose to sacrifice brightness in order to obtain pulses with vastly larger numbers of electrons than from the emitter if for a given application the number of electrons is a crucial figure of merit. We believe that our straightforward and flexible design will be of great practical relevance to researchers wishing to enter the field.

## I. INTRODUCTION

Time-resolved electron microscopy has proven to be a powerful tool for the study of the fast dynamics of nanoscale systems. The versatility of the technique is underlined by the vast range of phenomena that have been investigated, including mechanics,^1–5^ fluid dynamics,^6^ phase transitions,^7,8^ chemical reactions,^9–11^ the dynamics of magnetic structures,^12^ or the visualization of optical near fields.^13^ The various implementations of the technique have in common that sample dynamics are initiated *in situ* with a fast trigger, which are then probed at a well-defined point in time with a short electron pulse, such as to capture an image, diffraction pattern, or energy loss spectrum. In such a pump-probe experiment, the time resolution is no longer determined by the speed of the electron camera, but instead by the duration of the sample excitation pulse and the electron probe pulse. Crucially, the temporal resolution of time-resolved electron microscopy can, thus, be matched to the inherent timescale of atomic-scale motions in a wide range of processes from microseconds to femtoseconds and even attoseconds.^14–16^

Along with the increasing interest in time-resolved electron microscopy, the development of instruments has been burgeoning, and a range of new technologies are being pursued to improve their operation. This includes different approaches of generating electron pulses, either through photoemission from the filament through illumination with a short laser pulse or by chopping a continuous electron beam into pulses with a beam blanker,^17^ located either before^18–20^ or after the sample.^21,22^ Another active area of development is the generation of ultrafast electron pulses with high bunch charges, either through pulse compression with radio frequency cavities^23–26^ and THz laser pulses^27^ or by accelerating electrons to MeV energies.^28,29^ The importance of the choice of the electron source has also come into focus as it crucially determines the properties of the electron pulses. While flat metal photocathodes and LaB_6_ emitters are most widely used,^30–35^ field emitters have recently begun to advance novel types of time-resolved experiments.^21,22,36–38^ Their high brightness and coherence,^39–43^ which makes them the electron source of choice in most high-performance transmission electron microscopes, have enabled time-resolved electron holography experiments^21,36,44^ and have greatly advanced time-resolved experiments with fine electron probes.^14,15^ Moreover, they stand to significantly benefit time-resolved imaging at atomic resolution.^17,45^ At the same time, however, the small source size of field emitters also limits the number of electrons that can be extracted in a pulse, putting them at a disadvantage for applications in which the number of electrons is a critical figure of merit. For example, in a single-shot experiment, in which an irreversible process has to be captured with just a single electron pulse,^30,31^ the crucial challenge frequently lies in overcoming the shot noise. Clearly, if the available number of electrons is insufficient, capturing an event becomes unfeasible, no matter the brightness of the pulse. Such experiments are, therefore, typically performed with pulses containing millions of electrons^28,31,46^ that are generated through photoemission from flat emitters.

Here, we describe a straightforward design of a time-resolved transmission electron microscope with a Schottky field emission gun^47–49^ that offers operation both with high-brightness electron pulses from the emitter tip and with pulses containing a large number of electrons as if the microscope was equipped with a flat photocathode. We provide details of our design, in which the emitter is illuminated under a small angle with respect to the electron optical axis, and demonstrate that it affords a high spatial, temporal, and energy resolution as well as a high transverse brightness in pulsed operation. The geometry of our electron gun also allows us to create photoelectron pulses from the surface of the extractor instead of the emitter. We demonstrate that it is, thus, possible to extract vastly larger numbers of electrons per pulse and, thus, effectively trade brightness for electron counts, depending on the desired application.^48^

## II. EXPERIMENTAL SETUP

**FIG. 1.**
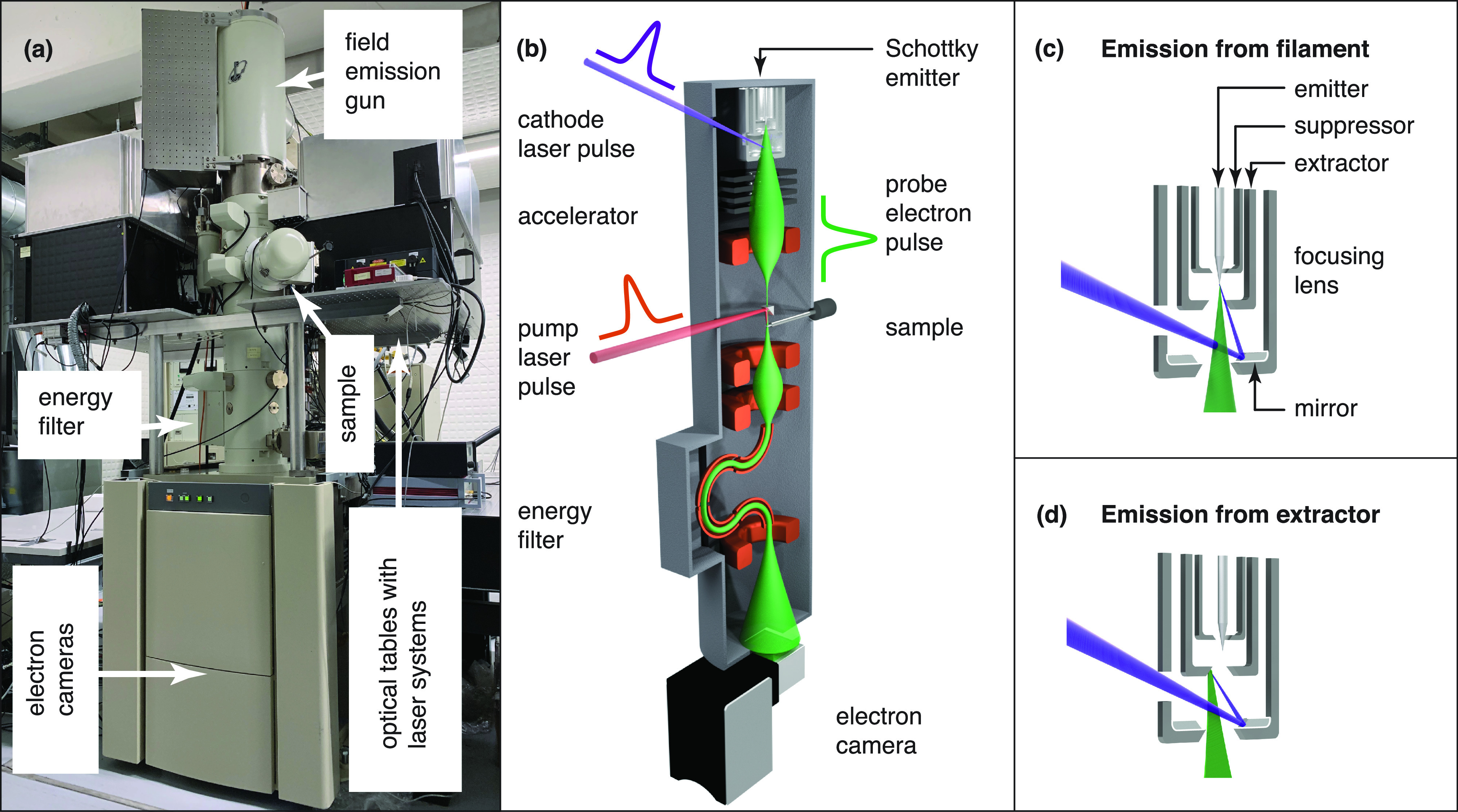
Overview of the time-resolved electron microscope. (a) Photograph of the JEOL 2200FS transmission electron microscope in the laboratory. (b) Sketch of the modified instrument. (c) Illustration of the modified Schottky emitter assembly. Photoelectron pulses of high brightness are generated by illuminating the tip of the emitter with UV laser pulses. (d) Pulses with large numbers of electrons are obtained by illuminating the extractor.

**FIG. 2.**
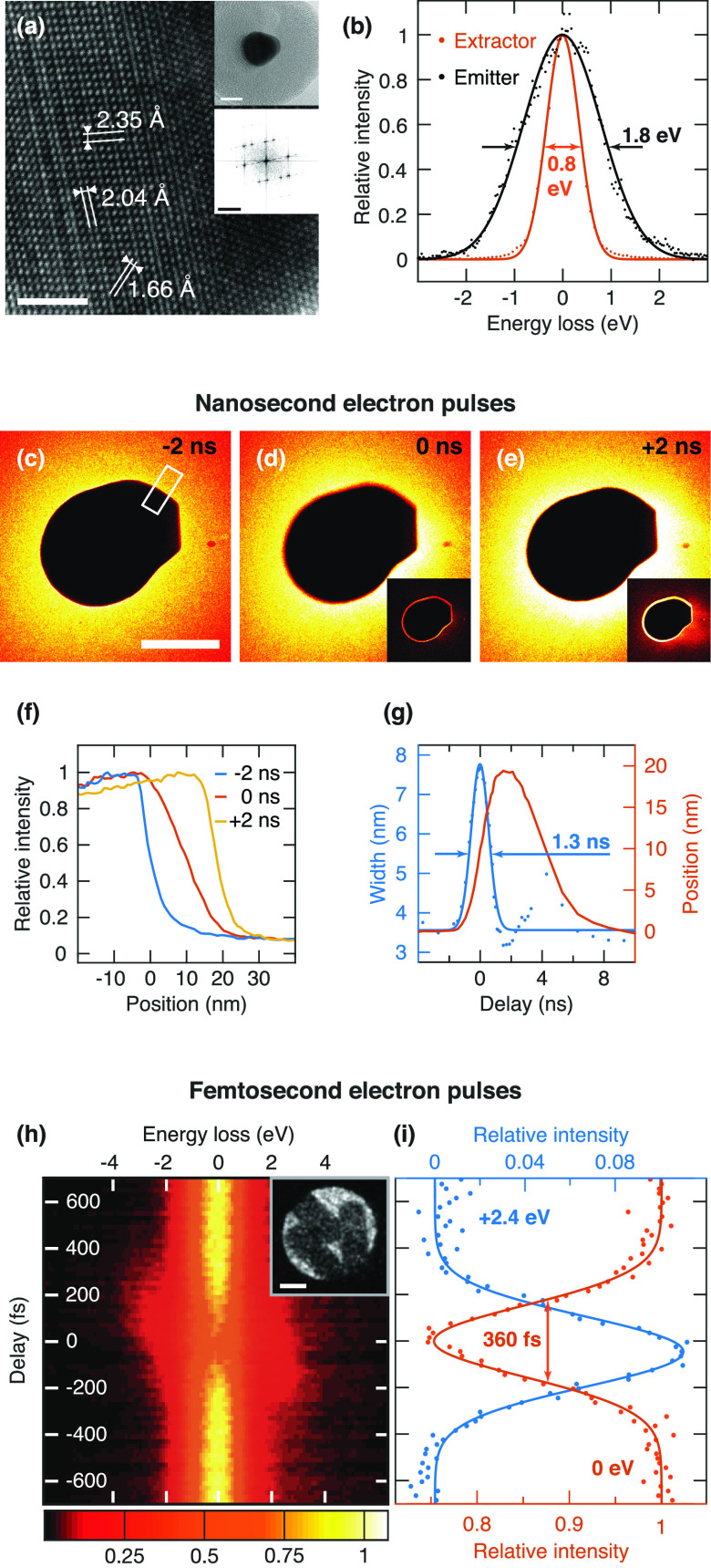
Characterization of the spatial, energy, and temporal resolution. (a) High resolution image of the core of the gold-core silica-shell nanoparticle shown in the top inset. The bottom inset displays the corresponding diffractrogram. Scale bar, 2 nm (10 and 5 nm^−1^ in the insets). (b) Energy distribution of photoelectrons from the filament and the extractor. Gaussian fits (solid lines) yield a FWHM of 1.8 eV and 0.8 eV, respectively. (c)-(e) Determination of the duration of nanosecond electron pulses from images of a gold nanoparticle under irradiation with a femtosecond laser pulse at different time delays. The insets display the difference with the image at negative time delay. Scale bar, 100 nm. (f) Intensity profiles of the boundary of the particle for different time delays, calculated from the area marked in (c). (g) The width and center position of the particle boundary are determined from a fit with an error function and displayed as a function of time. A Gaussian fit of the width yields an electron pulse duration of 1.3 ns. [(h) and (i)] Determination of the duration of femtosecond electron pulses. (h) Temporal evolution of the energy loss spectra collected from an ensemble of gold nanorods (inset) under femtosecond laser irradiation. Scale bar of the inset, 50 nm. (i) Relative intensity of the zero loss peak (0 eV, red) and of a sideband (+2.4 eV, blue) as a function of time (2 eV integration window). Gaussian fits (solid lines) yield a FWHM of 360 fs, corresponding to an electron pulse duration of 320 fs.

Figure 1 provides an overview of our modified JEOL JEM-2200FS transmission electron microscope, with a photograph in Fig. 1(a) and a schematic of the operating principle in Fig. 1(b). The remote operated microscope is equipped with a 200 kV Schottky field emission gun and an in-column Omega-type energy filter. A scintillator based electron camera (Gatan Ultrascan 4000) was used to record the majority of the data in this manuscript, while the high resolution image in Fig. 2(a) was acquired with a direct electron detector (Gatan K3). As shown in the photograph of Fig. 1(a), we mounted the entire laser system directly onto the microscope. This allows us to minimize the beam paths and use the same vibration damping system for the microscope and the lasers, so that more stable operation can be achieved.^34^ In order to provide sufficient space for the lasers and optics, we mounted two levels of breadboards on either side of the column. In the photograph, the optical setup is hidden behind enclosures that protect it from dust. The output of a femtosecond laser (Light Conversion Pharos, 190 fs laser pulses at 1032 nm, up to 1 MHz repetition rate) is doubled to excite the sample (516 nm, 160 fs), while the fourth harmonic (258 nm) is used to illuminate the emitter and create ultrafast photoelectron pulses. In order to access longer timescales, we generate nanosecond electron pulses with an Innolas Picolo 50 MOPA (1 ns pulses at 266 nm, up to 50 kHz repetition rate). Here, the ultrafast and nanosecond experiments were performed at 100 and 20 kHz repetition rate, respectively.

Figure 1(b) illustrates the operating principle of the time-resolved electron microscope. Sample dynamics are initiated *in situ* with a pump laser pulse,^47^ which is directed at the sample by means of an aluminum mirror that is mounted above the upper pole piece of the objective lens (Integrated Dynamic Electron Solutions) and that steers the laser beam straight down, so that it propagates almost collinearly with the electron beam.^50^ A 250 mm lens focuses the laser beam to a spot size of 24 *μ*m full width at half maximum (FWHM) in the sample plane as determined by a knife edge scan. In order to generate probe electron pulses, we cool the emitter to about 1100–1300 K, so that the continuous emission current becomes negligible and illuminate the tip of the Schottky emitter with UV laser pulses. The resulting photoelectron pulses are accelerated to 160 keV, interact with the sample, and after passing the energy filter, are detected by the electron camera. Since our electron gun is a refurbished model, we currently limit its accelerating voltage to 160 kV. However, we note that we have since modified an identical field emission gun that we are operating at 200 kV on a JEOL JEM-2010F.^49^

In order to generate photoelectron pulses, the emitter is illuminated through a viewport on the side of the gun that ordinarily serves for electron beam monitoring purposes. As illustrated in Fig. 1(c), the laser beam enters the emitter assembly through a hole in the electrostatic focusing lens.^49^ It is reflected by an aluminum mirror and strikes the tip of the Schottky emitter (York Probe Sources, 400 nm tip radius) under an angle of 16° with respect to the electron optical axis. The laser beam is focused onto the emitter by means of 125 mm lens which is located within the vacuum chamber of the gun. We typically apply +4 kV to the extractor (680 *μ*m aperture), and bias the suppressor to −300 V with respect to the emitter, while the focusing lens is operated at +6.0 kV.^49^ While illuminating the emitter allows us to generate high brightness electron pulses, the small source size also limits the number of electrons that can be extracted per pulse. Alternatively, we can create photoelectrons by directing the UV laser at the extractor [Fig. 1(d)]. The source size can then be adjusted by changing the spot size of the laser, which we can focus as tightly as 25 *μ*m FWHM. In this configuration, the gun essentially operates as if equipped with a flat photocathode or thermionic emitter, with the larger source size resulting in a vastly greater number of electrons per pulse, albeit at lower brightness. Here, we operate with the original extractor, which is made of stainless steel. In our second instrument, we have replaced it with an extractor machined out of copper, a more frequently used photocathode material.^51,52^

## III. RESULTS AND DISCUSSION

We begin by characterizing the spatial, energy, and temporal resolution of the time-resolved electron microscope. Figure 2(a) shows a high-resolution micrograph of the gold core of a silica-shell gold-core nanoparticle (top inset, 20 nm core diameter and 20 nm shell thickness) that was recorded with a continuous electron beam. The visibility of lattice fringes (see also the diffractogram in the bottom inset) demonstrates that our modifications have not deteriorated resolving power of the instrument. Figure 2(b) shows energy loss spectra of nanosecond photoelectron pulses that were obtained by illuminating either the emitter (black dots) or the extractor (red dots). Gaussian fits (solid lines) yield a FWHM of 1.8 eV and 0.8 eV, respectively. The difference in energy spread results from the difference in work function of the emitting surface. While the work function of the stainless steel surface of the extractor^53^ closely matches the photon energy (4.8 eV), the zirconium oxide coated tungsten emitter has a work function of only 2.8 eV.^54^ Therefore, photoelectrons are emitted with considerable excess energy, which leads to the larger energy spread.^55^

We characterize the temporal resolution for experiments with nanosecond electron pulses by making use of the transient electric field effect.^35,56,57^ Figure 2(c) shows a 200 nm diameter gold particle on a multilayer graphene substrate, imaged with nanosecond electron pulses from the emitter. Under irradiation with a femtosecond laser pulse, the sample emits a cloud of electrons, which deflects the probe electrons, so that images recorded at zero time delay or at small positive times appear distorted [Figs. 2(d) and 2(e)]. This is highlighted by the difference images displayed in the insets, which are obtained by subtracting the image at negative time delay [Fig. 2(c)]. To analyze the observed image distortions, we calculate intensity profiles across the particle border [Fig. 2(c), white rectangle], which reveal that the boundary shifts with time and that it broadens around time zero [Fig. 2(f)]. By fitting with an error function, we determine the position and the width of the particle edge as a function of time delay [Fig. 2(g), red line and blue dots, respectively]. The blurring of the particle boundary arises as the emergence of the electron cloud deflects the probe electron pulse,^35,56,57^ a process that occurs on a picosecond timescale, much faster than the duration of the electron pulse. We can, therefore, use this blurring to estimate the electron pulse duration, for which we obtain 1.3 ns from a fit of the boundary width with a Gaussian function (blue solid line).

We determine the femtosecond electron pulse duration through cross correlation of the electron and laser pulse.^13,58^ To this end, we use the fact that probe electrons interact inelastically with the scattered near-fields of a nanostructure under laser illumination, causing the electrons to gain or lose energy in multiples of the photon energy. Figure 2(h) displays the evolution of the energy loss spectrum of three gold nanorods (inset) under illumination with the pump laser (15 nJ pulse energy) as a function of time delay. When the electron and laser pulse overlap in time, the intensity of the zero loss peak decreases, while sidebands at ±2.4 eV, the photon energy, appear. Figure 2(i) shows the evolution of the intensity of the zero loss peak (red dots) and the sideband at +2.4 eV (blue dots). From Gaussian fits (solid lines), we extract a FWHM of the interaction time of 360 fs, which yields an electron pulse duration of 320 fs. We note that in this measurement we have reduced the number of electrons to less than one per pulse [as counted in the sample plane without a condenser lens aperture (CLA) inserted] in order to obtain the shortest pulse duration. The effects of space charge on the electron pulse properties are described in the following.

**FIG. 3.**
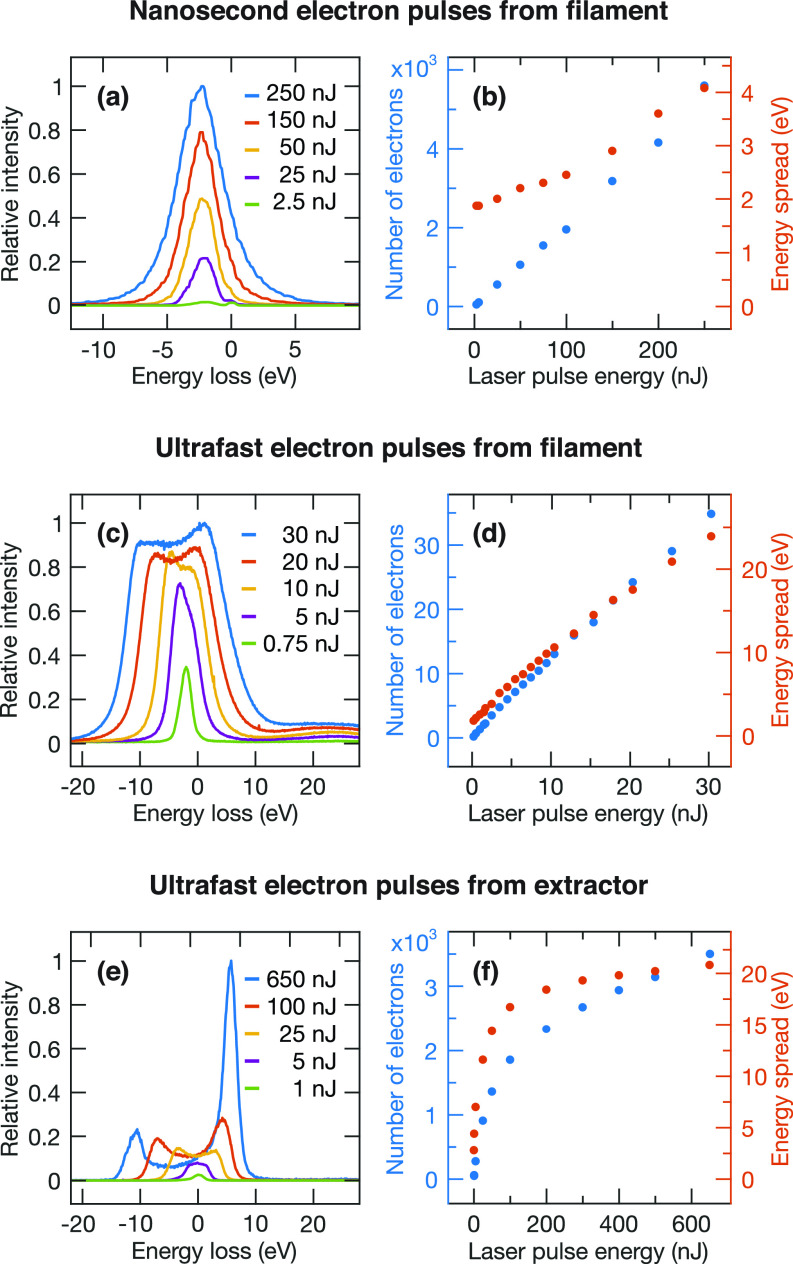
Characterization of electron pulses with high charge densities. Energy loss spectra are shown together with the electron yield and the energy spread as a function of laser pulse energy for [(a) and (b)] nanosecond electron pulses from the emitter, [(c) and (d)] ultrafast electron pulses from the emitter, and [(e) and (f)] ultrafast electron pulses from the extractor. The number of electrons is determined in the sample plane (without condenser lens aperture inserted). The energy spread is measured as the FWHM of the distribution. For distributions featuring two maxima, we report the full width at half the height of the smaller maximum.

In Fig. 3, we characterize the generation of pulses with high charge densities, which is of particular relevance for applications in which the available number of electrons is the most important figure of merit. We begin by studying the evolution of the energy loss spectra for nanosecond electron pulses from the emitter as a function of the pulse energy of the cathode laser [Fig. 3(a)]. With increasing pulse energy, the number of photoelectrons per pulse grows, and the increasing space charge repulsion within the electron packet broadens its energy distribution.^59^ We note that due to the excess energy with which the photoelectrons are emitted, their energy distribution is shifted by about 2 eV with respect to that of the continuous electron beam.^60,61^ This is evident in the spectra recorded at low pulse energies, which exhibit some residual continuous emission that is apparent as a second peak centered at 0 eV. As shown in Fig. 3(b), we find that the number or electrons per pulse increases linearly with the laser pulse energy (blue dots) and reaches over 5000 in the sample plane (no condenser lens aperture) at 250 nJ, while the FWHM energy spread increases from 2 eV to 4 eV (red dots).

Ultrafast electron pulses from the filament are even more strongly affected by space charge [Figs. 3(c) and 3(d)]. As we raise the laser pulse energy to 30 nJ, which increases the number of electrons per pulse to 35, the energy spread reaches almost 25 eV [Fig. 3(d)], with the energy distribution featuring a characteristic double peak structure [Fig. 3(c)].^59,62,63^ Evidently, the number of electrons per pulse can only be increased significantly by sacrificing the energy resolution. Moreover, the larger energy spread of the electrons couples to the chromatic aberration of the electron optical system. It also broadens the arrival time distribution of the electrons at the sample, thus increasing the electron pulse duration and lowering the time resolution of the experiment to several picoseconds, as we will discuss in more detail below.

Significantly larger numbers of electrons per pulse with a comparable energy spread can be obtained by illuminating the surface of the extractor electrode instead of the tip of the emitter [Figs. 3(e) and 3(f)]. The number of electrons emitted from the extractor initially increases rapidly with the laser pulse energy, but begins to level off above 100 nJ, finally reaching 3500 electrons at 650 nJ [Fig. 3(f), blue dots]. The energy spread increases to 20 eV at 200 nJ pulse energy, after which it grows only marginally (red dots). For large numbers of electrons per pulse, the energy loss spectra feature a pronounced double peak structure [Fig. 3(e)]. For pulses of such high bunch charge, the pulse duration stretches to several tens of picoseconds.

**FIG. 4.**
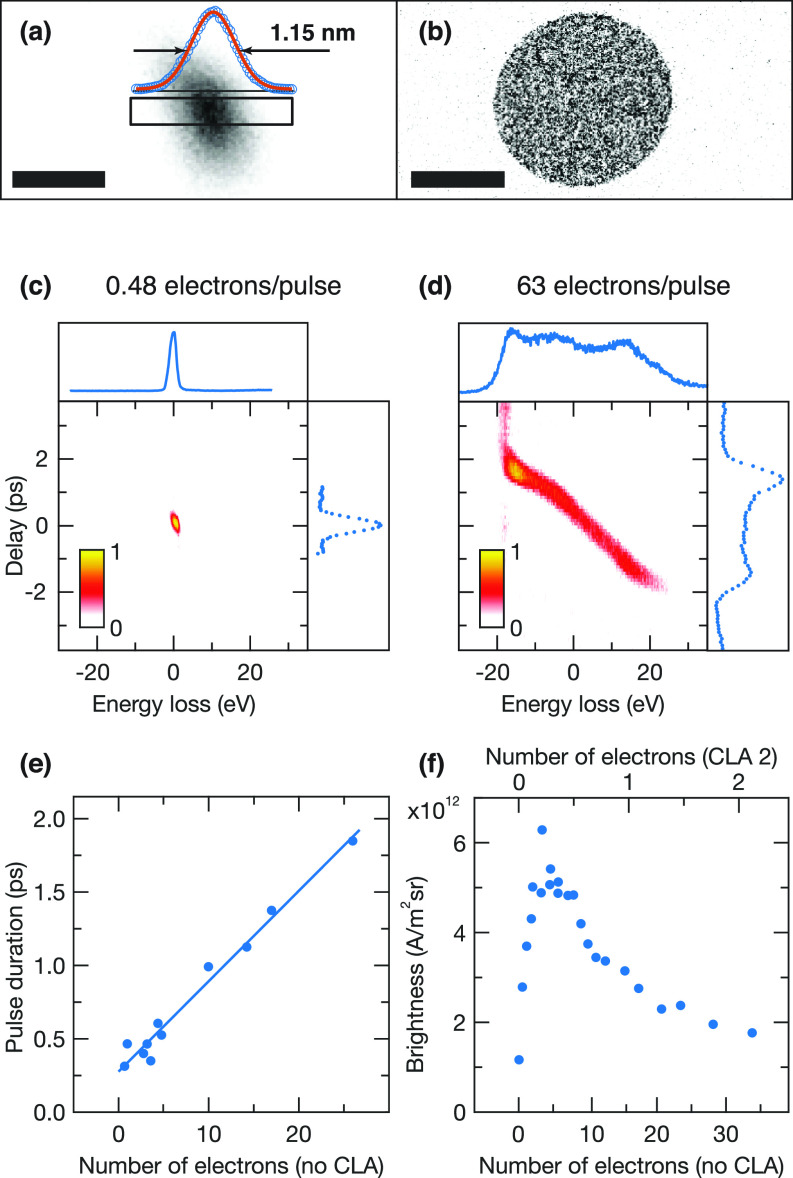
Determination of the brightness for ultrafast electron pulses. (a) Image of the converged electron beam (convergent beam electron diffraction mode) with a spot size of 1.15 nm, as determined from a Gaussian fit (red line) of an intensity profile (blue dots) of the area marked with a black rectangle. Scale bar, 2 nm. (b) Diffraction pattern of the converged electron beam. Scale bar, 2 mrad. [(c) and (d)] Time-energy distributions of electron pulses with 0.48 and 63 electrons per pulse, respectively. The projections of the pulses into the time and energy domains are presented on the top and on the right, respectively. (e) Pulse duration as a function of the number of electrons per pulse [spot size 1, alpha 3, no condenser lens aperture (CLA) inserted]. For pulses with two maxima in the time domain, we report the full width at half the height of the smaller maximum. A linear fit serves as a guide for the eye. (f) Brightness as a function of the number of electrons per pulse (spot size 1, alpha 3, CLA 2). The brightness is measured with the second condenser lens aperture inserted, which reduces the number of electrons in the sample plane by a factor of about 15. For easy comparison with (e), we report the number of electrons without condenser lens aperture inserted on the bottom axis and the actual number of electrons on the top axis.

Finally, we demonstrate that our gun design achieves a brightness that is comparable to that of other time-resolved field emission gun microscopes. The transverse brightness of the electron beam can be conveniently determined at a beam waist, which can be formed by focusing the electron beam in the sample plane.^19^ In such a configuration, the instantaneous brightness B of the electron pulse can then be obtained as the instantaneous probe current Ne/Δt per surface area element $\pi r^{2}$ and solid angle $\pi \alpha^{2}$,

$$B=\frac{Ne/\Delta t}{\pi^{2}r^{2}\alpha^{2}},$$

where N is the number of electrons per pulse, e is the electron charge, Δt is the pulse duration, r is the spot radius, and α is the convergence semi-angle of the beam. To illustrate the measurement principle, Fig. 4(a) shows a micrograph of the focused electron beam, with the microscope operated in convergent beam electron diffraction mode. From an intensity profile (blue circles) of the area marked with a black rectangle, we obtain a FWHM spot size of 2r= 1.15 nm, as determined from a fit with a Gaussian (red line). The spot can be seen to be slightly asymmetric. In the following, we, therefore, determine the spot radius according to $r=\sqrt{r_{x}r_{y}}$, with $r_{x}$ and $r_{y}$ the semi-axes of the ellipse. The diffraction pattern corresponding to the beam in (a) is displayed in Fig. 4(b), from which we determine a convergence semi-angle α of 3.7 mrad.

Figures 4(c) and 4(d) illustrates the determination of the electron pulse duration from time-energy profiles. These are obtained by recording energy loss spectra of the inelastic electron–photon interactions at a nanostructure under laser illumination [e.g., Fig. 2(h)] and plotting difference spectra as a function of time. Projections of the pulses into the energy and time domain are shown on top of the figure and on its right, respectively. The shortest pulse durations and most narrow energy distribution are obtained for pulses with a small number of electrons. For example, in Fig. 4(c), pulses containing 0.48 electrons on average (counted in the sample plane) yield a duration of 410 fs and an energy spread of 2.1 eV FWHM. For large numbers of electrons per pulse, space charge repulsion broadens both distributions, as shown in Fig. 4(d) for pulses with 63 electrons on average, which have a pulse duration of 3800 fs and an energy spread of 39.4 eV. Such pulses possess a distinct shape in the time-energy distribution with a large central portion that has a constant chirp as well as smaller wings. Figure 4(e) shows that the pulse duration that we extract from the time-energy diagrams increases linearly with the number of electrons per pulse. For pulses with two maxima in the time domain, such as in Fig. 4(d), we report the pulse length as the full width at half the height of the smaller maximum.

With the above determined quantities, we calculate the instantaneous brightness as a function of the number of electrons per pulse [Fig. 4(f)]. Here, the brightness is measured with the second condenser lens aperture inserted, which reduces the number of electrons in the sample by about a factor of 15. For easy comparison with Fig. 4(e), we report the number of electrons that would be obtained without condenser lens aperture on the bottom axis and the actual number of electrons on the top axis. The brightness initially increases linearly with the number of electrons, but it begins to level off as electron–electron interactions become more frequent, causing the spot size to grow and the pulse duration to increase. The brightness reaches a maximum value of 6.3 × 10^12^ A/(m^2^ sr) at 0.23 electrons per pulse (in the sample plane) before decreasing again for even larger numbers of electrons. This behavior resembles that of continuous electron beams from field emitters, whose brightness initially grows with increasing emission current, but then levels off as electron–electron interactions become more frequent^64^ and finally goes through a maximum.^65^ Typical values for the instantaneous brightness are given in Table I for different operating parameters of the microscope. Notably, the instantaneous brightness of the femtosecond electron pulses is higher than that of our continuous electron beam (Table I, first line) and similar to that of side-illuminated field emitters.^36,37^ The brightness can be further increased by raising the accelerating voltage to 200 kV, as we have done on our second time-resolved instrument, choosing a smaller emitter size, and matching the photon energy of the cathode laser more closely to the work function of the emitter. Table I also includes a brightness measurement for electron pulses generated from the extractor. While the larger emission area on the extractor allows us to obtain higher photocurrents than from the small tip of the emitter [Fig. 3(f)], the larger source size also inevitably reduces the brightness of the beam by more than two orders of magnitude.^17^

**TABLE I.** Brightness of the photoelectron pulses for different operating parameters. For a given laser pulse energy, settings of the condenser lens system (Spot size and Alpha), and condenser lens aperture number (CLA), the table lists the number of electrons per pulse in the sample plane (*N*), the pulse duration (Δ*t*), the instantaneous probe current (*I*), the FWHM of the focused electron beam (*d*), the convergence semi-angle (*α*), and the instantaneous brightness (*B*). Unless otherwise noted, electron pulses are generated by illuminating the emitter. For the duration of the nanosecond electron pulses, we assume 1 ns, the duration of the cathode laser pulses.

Laser pulse energy and duration	Spot size	Alpha	CLA	N	Δt (ps)	I (nA)	d (nm)	*α* (mrad)	B (A/m^2^ sr)
Continuous beam	5	3	3	…	…	0.105	1.8	3.7	9.0 × 10^11^
30 nJ, fs	5	3	3	0.026	0.45	9.3	7.3	3.7	5.1 × 10^12^
2 nJ, fs	1	3	2	0.23	0.48	76	7.5	9.3	6.3 × 10^12^
10 nJ, fs	1	3	2	0.79	0.83	117	12.8	9.3	3.3 × 10^12^
30 nJ, fs	1	3	2	2.05	1.90	143	19.5	9.3	1.8 × 10^12^
300 nJ, ns	1	3	2	219	1000	35	10.1	9.3	1.6 × 10^12^
150 nJ, ns (extractor)	1	3	2	111	1000	17.7	133	9.3	4.7 × 10^9^

## IV. CONCLUSION

We expect that our straightforward design of a time-resolved transmission electron microscope with a field emission gun will facilitate the adoption of these instruments, which are more challenging to modify and operate, but they are opening up new possibilities for studying the fast dynamics of nanoscale systems. We demonstrate that our design with a front-illuminated emitter achieves a high spatial, energy, and temporal resolution as well as a brightness that is similar to that of designs in which the emitter is illuminated from the side. Alternatively, the instrument can be operated as if it was equipped with a flat photocathode by creating photoelectrons from the extractor instead of the emitter tip. It thus becomes possible to trade brightness for counts, if this is advantageous for a given application. In our lab, we can switch between both operating modes within minutes, thanks to an optical setup with flip mirrors. We believe that this flexibility will also be useful for the integration of pulse compression cavities into time-resolved microscopes.^26^ If the purpose of the cavity is to compress single-electron pulses and thus improve the time resolution of the experiment,^66^ creating electrons from the tip of the emitter will be preferable due to the smaller source size. However, if the cavity is instead used to boost sensitivity by increasing the number of electrons per pulse without sacrificing time resolution, the number of electrons from the emitter may not be sufficiently high, and creating a large number of electrons from the extractor can be advantageous. Our characterization of space-charge limited electron pulses from the emitter and extractor offers a guide for choosing the best operating parameters for a given experiment.

## Data Availability

The data that support the findings of this study are available from the corresponding author upon reasonable request.
